# Survival, but not the severity of hypoxic–ischemic encephalopathy, is associated with higher mean arterial blood pressure after cardiac arrest: a retrospective cohort study

**DOI:** 10.3389/fcvm.2024.1337344

**Published:** 2024-05-07

**Authors:** Sandra Preuß, Jan Multmeier, Werner Stenzel, Sebastian Major, Christoph J. Ploner, Christian Storm, Jens Nee, Christoph Leithner, Christian Endisch

**Affiliations:** ^1^ Department of Neurology, AG Emergency and Critical Care Neurology, Campus Virchow Klinikum, Charité Universitätsmedizin Berlin, Berlin, Germany; ^2^ Department of Cardiology and Angiology, Charité Campus Mitte, Charité Universitätsmedizin Berlin, Berlin, Germany; ^3^ Ada Health GmbH, Berlin, Germany; ^4^ Department of Neuropathology, Charité Campus Mitte, Charité Universitätsmedizin Berlin, Berlin, Germany; ^5^ Center for Stroke Research, Charité Universitätsmedizin Berlin, Berlin, Germany; ^6^ Department of Nephrology and Intensive Care Medicine, Cardiac Arrest Center of Excellence Berlin, Campus Virchow Klinikum, Charité Universitätsmedizin Berlin, Berlin, Germany

**Keywords:** cardiac arrest (CA), brain autopsy, hypoxic–ischemic encephalopathy (HIE), mean arterial pressure (MAP), cumulative vasopressor index, prognosis

## Abstract

**Background:**

This study investigates the association between the mean arterial blood pressure (MAP), vasopressor requirement, and severity of hypoxic–ischemic encephalopathy (HIE) after cardiac arrest (CA).

**Methods:**

Between 2008 and 2017, we retrospectively analyzed the MAP 200 h after CA and quantified the vasopressor requirements using the cumulative vasopressor index (CVI). Through a postmortem brain autopsy in non-survivors, the severity of the HIE was histopathologically dichotomized into no/mild and severe HIE. In survivors, we dichotomized the severity of HIE into no/mild cerebral performance category (CPC) 1 and severe HIE (CPC 4). We investigated the regain of consciousness, causes of death, and 5-day survival as hemodynamic confounders.

**Results:**

Among the 350 non-survivors, 117 had histopathologically severe HIE while 233 had no/mild HIE, without differences observed in the MAP (73.1 vs. 72.0 mmHg, *p*_group _= 0.639). Compared to the non-survivors, 211 patients with CPC 1 and 57 patients with CPC 4 had higher MAP values that showed significant, but clinically non-relevant, MAP differences (81.2 vs. 82.3 mmHg, *p*_group _< 0.001). The no/mild HIE non-survivors (*n* = 54), who regained consciousness before death, had higher MAP values compared to those with no/mild HIE (*n* = 179), who remained persistently comatose (74.7 vs. 69.3 mmHg, *p*_group _< 0.001). The no/mild HIE non-survivors, who regained consciousness, required fewer vasopressors (CVI 2.1 vs. 3.6, *p*_group _< 0.001). Independent of the severity of HIE, the survivors were weaned faster from vasopressors (CVI 1.0).

**Conclusions:**

Although a higher MAP was associated with survival in CA patients treated with a vasopressor-supported MAP target above 65 mmHg, the severity of HIE was not. Awakening from coma was associated with less vasopressor requirements. Our results provide no evidence for a MAP target above the current guideline recommendations that can decrease the severity of HIE.

## Background

The severity of hypoxic–ischemic encephalopathy (HIE) mainly determines the long-term outcomes in comatose patients resuscitated from cardiac arrest (CA) (1–5). Hemodynamic instability contributes to increased mortality and secondary brain injury (6–8). Blood pressure management with vasopressors aims to maintain sufficient cerebral perfusion and avoid secondary ischemia as cerebral blood flow autoregulation is impaired following cerebral ischemia (9–11).

Current international guidelines recommend the avoidance of having a mean arterial blood pressure (MAP) below 65 mmHg. Vasopressors are recommended to counteract lower values, but evidence supporting a specific MAP target is lacking (11, 12). Hypotension and increased vasopressor requirements were previously found to be associated with increased mortality and poor neurological outcomes (13, 14). However, interventional studies comparing different MAP targets failed to demonstrate improved outcomes with a MAP above 65 mmHg (15–18). Importantly, extracerebral death causes are frequent in patients without severe HIE and confound studies with mortality as the primary outcome (1, 3).

Hence, we studied the association between the MAP, vasopressors, and severity of HIE evaluated by quantified histopathology and neurological status considering death causes.

## Methods

### Study design and post-resuscitation care

The ethical committee of the Charité University Hospital approved the study (approval number: EA2/007/16; title: “Prognosis after cardiac arrest and resuscitation—Evaluation of prognostic parameters using brain autopsy”) on 5 February 2016 and waived the need for patient consent. The study complied with the 1975 Helsinki Declaration and was written following the Strengthening the Reporting of Observational Studies in Epidemiology guidelines. In the non-survivor cohort, we retrospectively included consecutively admitted patients with postmortem brain autopsies after an initially successful resuscitation from all intensive care units (ICU) at the Charité University Hospital in Berlin, Germany, between January 2008 and May 2017. Treatment adhered to the international guidelines of post-resuscitation care (10, 19), including hemodynamic management to avoid a MAP below 65 mmHg and targeted temperature management (TTM) in 93 patients (27%) who underwent brain autopsy and 264 patients (99%) with ICU survival. We recorded the demographics following the Utstein-style guidelines (20), preexisting diseases, and temporary regain of consciousness (i.e., awake and communicating) before death. The clinical death causes were classified into cardiac cause, sepsis/multiorgan failure (MOF), brain injury, and others (1) based on the available clinical data. Neuroprognostication and withdrawal of life-sustaining therapy (WLST) followed international guidelines (10, 19). The treating physicians requested brain autopsies for a clinical quality check, independent of the study. In the survivor cohort, we included patients with coma or unresponsive wakefulness syndrome under cerebral performance category (CPC) 4 and those with no/mild neurological deficits (CPC 1) upon ICU discharge and treated in the ICU of the Cardiac Arrest Center of Excellence in Berlin between January 2006 and December 2015.

### Histopathological evaluation of postmortem brain autopsy

We histopathologically reevaluated and quantified neuronal death (ND) using the selective eosinophilic neuronal death (SEND) classification. The raters (SP and CE) were blinded from the clinical data, as previously described (21–24). Selective ND following cerebral reperfusion after resuscitation is visible as pyknotic, red neurons in contrast to necrosis that develops in unsuccessful resuscitation without cerebral reperfusion. In oxygen-sensitive regions (i.e., cortex, hippocampus, and cerebellum) and less oxygen-sensitive regions (i.e., basal ganglia, mesencephalon, pons, and medulla oblongata), we microscopically quantified selective eosinophilic ND ranging from 0% (SEND 0), below 30% (SEND 1), 30–60% (SEND 2), 60–90% (SEND 3), and above 90% (SEND 4). We histopathologically dichotomized the non-survivors into no/mild HIE and severe HIE (24).

### Hemodynamic monitoring

The invasively measured MAP was continuously monitored and stored in 30-min intervals. We calculated the mean and the interquartile ranges (IQR) hourly during the first 200 h. To quantify the vasopressor requirements, we determined the cumulative vasopressor index (CVI) hourly (25). The CVI numerically rates the dosages of dopamine, epinephrine, norepinephrine, phenylephrine, and vasopressin according to potency from 0 to 20, with higher values indicating higher vasopressor requirements (Supplementary Table S1). Stratifying the histopathological and clinical HIE severity, we studied the association between MAP and CVI. To consider hemodynamic biases, we investigated the regain of consciousness, death causes, selective histopathological damage patterns, and 5-day survival.

### Statistical analysis

The demographic variables are presented as median/mean and IQR, standard deviation (SD), or percentage and tested for statistical significance (*p* < 0.05) using the chi-squared test or Wilcoxon rank sum test. The histopathological results are illustrated as heatmaps. Continuous MAP and CVI data are presented as confidence plots and mutual dependence in scatter plots. We used a general linear model to test the statistical significance between groups (denoted as *p*_group_) and time, the interaction between groups and time during the first 200 h and between the first and second 100 h, and day-wise intervals. We quantitatively analyzed the results of the general linear model, reviewed the box plots, and provided descriptive statistics when suitable. Mutual MAP and CVI dependence was tested using Welch's two-sample *t*-test or Mann–Whitney *U* test for independent samples. For a multiple-group comparison, we compared pairwise with *p*-value correction for multiple testing through a Mann–Whitney *U* test. Analyses were performed using MATLAB Release 2019b (MathWorks, Inc.) and R Project Release 2020 (The R Project for Statistical Computing).

## Results

### Study population

**Figure 1:**
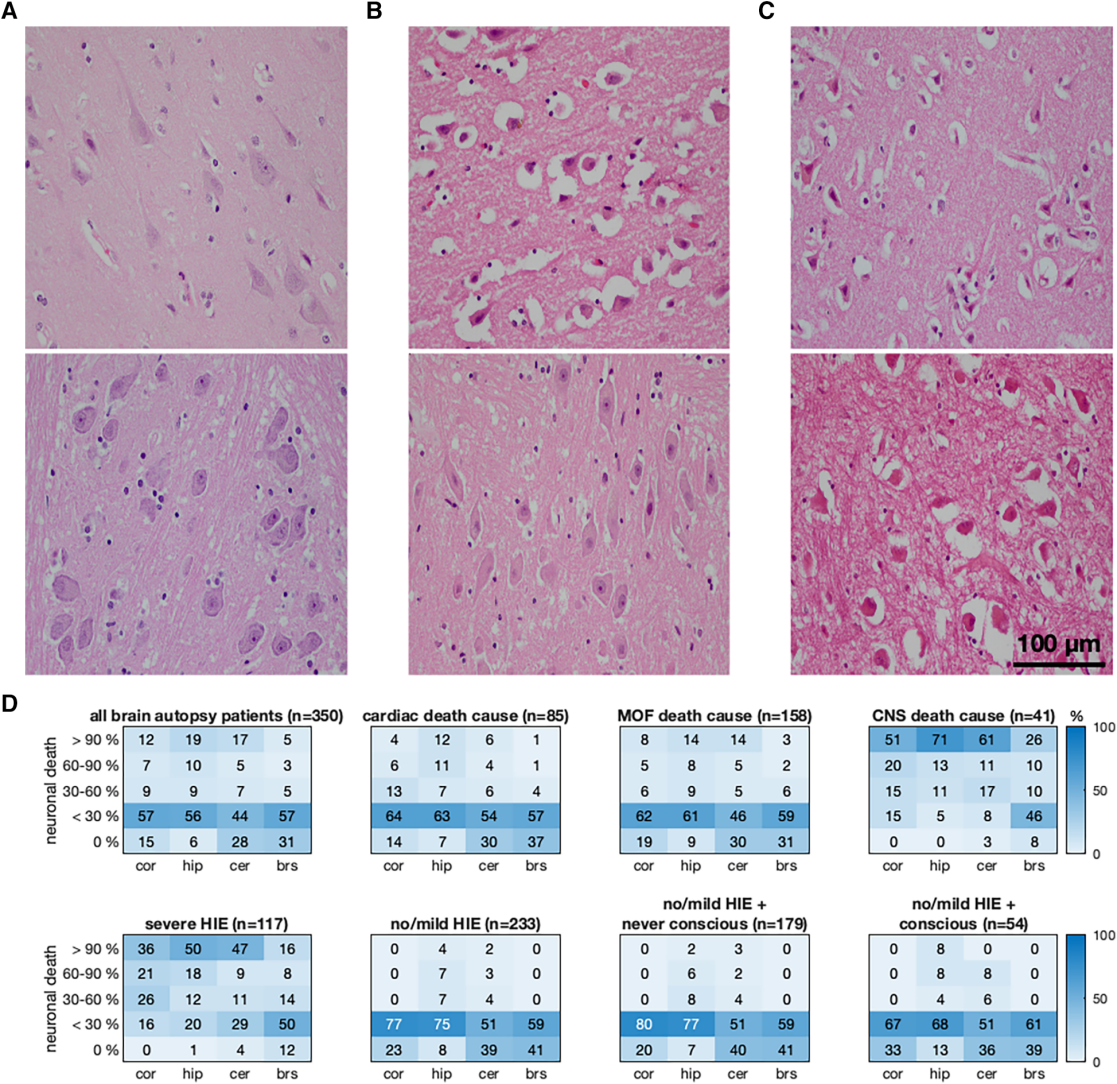
Association between the regional quantification of neuronal death (ND), regain of consciousness, and cause of death in non-survivors. The first two rows show typical hematoxylin and eosin-stained brain regions with cerebral cortical regions in the upper and pontine regions in the lower row. (**A**) No/mild HIE non-survivor without ND in the cerebral cortex and pons. (**B**) Severe HIE non-survivor with a cortical ND larger than 90% and nearly spared pons (ND below 30%). (**C**) Severe HIE non-survivor with a ND larger than 90% in the cerebral cortex and pons. (**D**) Quantitative histopathological severity of HIE according to the selective eosinophilic ND classification illustrated for the cerebral cortex, hippocampus, cerebellum, and brainstem (BS). The distributions of the HIE severity are presented as heatmaps (numbers and field color indicating percentage) for non-survivors and stratified by death causes, severe and no/mild HIE, and regain of consciousness (subheadings). MOF, multiorgan failure; CNS, central nerve system; HIE, hypoxic–ischemic encephalopathy; CA, cardiac arrest.

We included 618 patients, of whom 350 (57%) were non-survivors with brain autopsy and 268 (43%) were survivors. Table 1 shows the demographics, and Supplementary Table S2 presents the preexisting diseases. Among the non-survivors, 233 (67%) and 117 (33%) patients showed no/mild and severe HIE, respectively. Fifty-four (23%) of these 233 patients with no/mild HIE and 15 of the 117 (13%, *p* = 0.031) patients with severe HIE initially regained consciousness. The no/mild HIE non-survivors had more out-of-hospital CA (OHCA) (*p* < 0.001) and shorter resuscitations compared to the severe HIE non-survivors (10 min, IQR 5–23 vs. 15 min, 5–27, *p* = 0.017). Regain of consciousness in the no/mild HIE non-survivors was associated with shorter resuscitations (5 min, 1–10, *p* < 0.001). Of the 350 non-survivors, 85 (24%) had cardiac deaths; 158 (45%) had MOF; 41 (12%) had CNS death causes (Supplementary Tables S3, S4); and 66 (19%) had other death causes. Cardiac death patients had more preexisting cardiac diseases, whereas patients with no/mild HIE died primarily due to MOF (*p* = 0.002). Figure 1 illustrates the histopathological results and the regional distribution of ND stratified by death causes, HIE dichotomization, and regain of consciousness. In the survivor cohort, we included 211 (79%) CPC 1 patients and 57 (21%) CPC 4 patients. The CPC 4 patients had non-significantly longer resuscitations compared to the CPC 1 patients (15 min, 10–24 vs. 12 min, 8–20, *p* = 0.062). The CPC 1 patients were younger (*p* = 0.010) and had more cardiac CA causes (*p* = 0.004), more shockable rhythms (*p* = 0.003), and shorter ICU durations (*p* < 0.001).

**Table 1:** Patient characteristics.

Demographic characteristics	Severe HIE non-survivors (*n* = 117)	No/mild HIE non-survivors (*n* = 233)	Statistical comparison of severe HIE vs. no/mild HIE non-survivors, test statistic (*p*)^a^	CPC 4 survivors (*n* = 57)	CPC 1 survivors (*n* = 211)	Statistical comparison of the CPC 4 vs. CPC 1 survivors, test statistic (*p*)^a^
Gender, male (*n*, %)	69 (59)	145 (62)	χ^2 ^= 0.224 (*p* = 0.636)	35 (61)	163 (77)	χ^2 ^= 5.048 (*p* = 0.025)
Age, year (median, IQR)	68 (59–76)	69 (58–76)	W = 13,482 (*p* = 0.969)	62 (51–72)	59 (49–67)	W = 5,134 (*p* = 0.010)
OHCA (*n*, %)	75 (64)	215 (92)	χ^2 ^= 41.56 (*p* < 0.001)	45 (79)	159 (75)	χ^2 ^= 2.390 (*p* = 0.122)
Cardiac cause of CA (%)	36	31	χ^2 ^= 0.46 (*p* = 0.498)	58	78	χ^2 ^= 8.198 (*p* = 0.004)
Shockable initial rhythm (%)	30	23	χ^2 ^= 1.16 (*p* = 0.281)	36	71	χ^2 ^= 8.949 (*p* = 0.003)
tROSC, min (median, IQR)	15 (5–27)	10 (5–23)	W = 5,718 (*p* = 0.017)	15 (10–24)	12 (8–20)	W = 4,878.5 (*p* = 0.062)
Targeted temperature management (*n*, %)	53 (45)	40 (17)	χ^2 ^= 30.17 (*p* < 0.001)	54 (95)	210 (100)	χ^2 ^= 4.123 (*p* = 0.042)
Second CA with resuscitation during ICU stay (*n*, %)	49 (42)	108 (46)	χ^2 ^= 0.46 (*p* = 0.497)	Not applicable	Not applicable	-
Temporarily conscious during ICU stay (*n*, %)	15 (13)	54 (23)	χ^2 ^= 4.64 (*p* = 0.031)	Not applicable	Not applicable	-
WLST (*n*, %)	62 (53)	91 (39)	χ^2 ^= 5.59 (*p* = 0.018)	Not applicable	Not applicable	-
Length of ICU stay, day (median, IQR)	3 (1–8)	1 (0–8)	W = 11,026 (*p* = 0.003)	29 (21–38)	13 (8–23)	W = 3,037 (*p* < 0.001)
Death cause, cardiac (*n*, %)	*23* (*20)*	*62* (*27)*	χ^2 ^= 1.686 (*p* = 0.194)	Not applicable	Not applicable	-
Death cause, MOF (*n*, %)	*39* (*33)*	*119* (*51)*	χ^2 ^= 9.195 (*p* = 0.002)	Not applicable	Not applicable	-
Death cause, CNS (*n*, %)	*39* (*33)*	*2* (*1)*	χ^2 ^= 76.317 (*p* < 0.001)	Not applicable	Not applicable	-
Death cause, others (*n*, %)	*16* (*14)*	*50* (*22)*	χ^2 ^= 2.597 (*p* = 0.107)	Not applicable	Not applicable	-
Maximal SEND score (range 0–4), cortex (median, IQR)	3 (2–4)	1 (1–1)	W = 1,710 (*p* < 0.001)	Not applicable	Not applicable	-
Maximal SEND score (range 0–4), hippocampus (median, IQR)	3.5 (2–4)	1 (1–1)	W = 3,883.5 (*p* < 0.001)	Not applicable	Not applicable	-
Maximal SEND score (range 0–4), cerebellum (median, IQR)	3 (1–4)	1 (0–1)	W = 3,602.5 (*p* < 0.001)	Not applicable	Not applicable	-
Maximal SEND score (range 0–4), BS (median, IQR)	1 (1–2)	1 (0–1)	W = 6,338 (*p* < 0.001)	Not applicable	Not applicable	-

^a^ Categorical variables were compared using Chi-squared tests (χ^2^). Non-normally distributed continuous variables were compared using the Wilcoxon rank sum test (W).

HIE, hypoxic–ischemic encephalopathy; CPC, cerebral performance category; IQR, interquartile range; OHCA, out-of-hospital cardiac arrest; CA, cardiac arrest; tROSC, time from cardiac arrest to spontaneous circulation; ICU, intensive care unit; WLST, withdrawal of life-sustaining treatment; MOF, sepsis/multiorgan failure; CNS, clinically predicted severe brain injury; SEND, selective eosinophilic neuronal death; BS, brainstem.

### MAP and HIE severity

**Figure 2:**
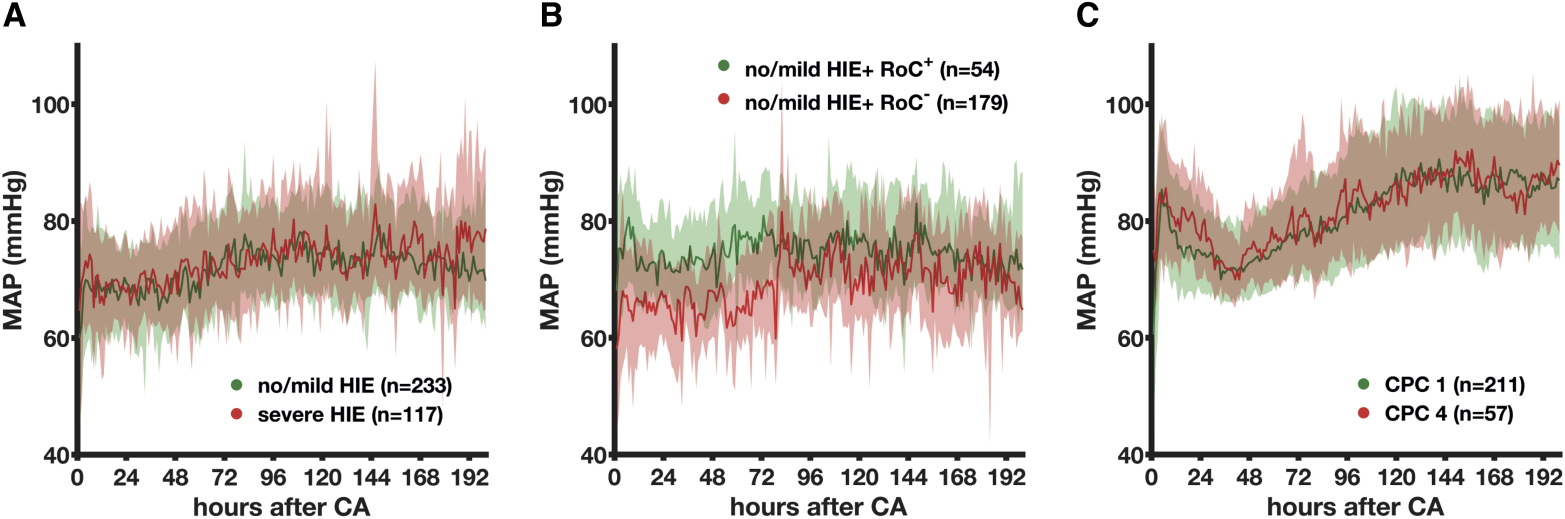
Association between the mean arterial blood pressure and the severity of hypoxic–ischemic encephalopathy evaluated by histopathology in non-survivors and neurological status in survivors. The MAP is presented here as median (bold line) and interquartile range (transparent area) depending on the histopathological severity of HIE (**A**), regain of consciousness (**B**), and clinical absence of HIE (**C**). MAP, mean arterial blood pressure; CA, cardiac arrest; HIE, hypoxic–ischemic encephalopathy; RoC^+^, regain of consciousness present; RoC^−^, never regained consciousness; CPC, cerebral performance category.

In the non-survivors, there was no difference in the MAP of patients with severe HIE and no/mild HIE (73.1 vs. 72.0 mmHg, *p*_group _ = 0.639). However, a significant change was observed over time (*p* < 0.001), with the MAP dropping below 60 mmHg in the first 48 h (Figure 2A). Compared to the 350 non-survivors, 211 CPC 1 patients and 57 CPC 4 patients had higher MAP values that differed (81.2 vs. 82.3 mmHg, *p*_group _< 0.001) and increased over time (*p* < 0.001) (Figure 2C). The mean MAP of the CPC 1 patients was 76.4 mmHg (80.5 mmHg in CPC 4) in the first 24 h. It was the lowest at 72.6 mmHg (73.8 mmHg in CPC 4) between 24 and 48 h and increased to a stable mean of 86.4 mmHg (86.5 mmHg in CPC 4) during the last 100 h. Except for the first 24 h, the MAP differences were not larger than 2 mmHg. The no/mild HIE non-survivors who regained consciousness had higher MAP values compared to those with persistent coma (74.7 mmHg vs. 69.3 mmHg, *p*_group _< 0.001), whose MAP values significantly increased over time (*p* < 0.001) (Figure 2B). The group comparison showed that the CPC 1 and CPC 4 patients had the highest mean MAP values of 81.2 and 82.3 mmHg, respectively, followed by the no/mild HIE non-survivors who regained consciousness (mean MAP, 74.7 mmHg; *p* < 0.001) during the first 200 h.

### Vasopressor therapy and HIE severity

**Figure 3:**
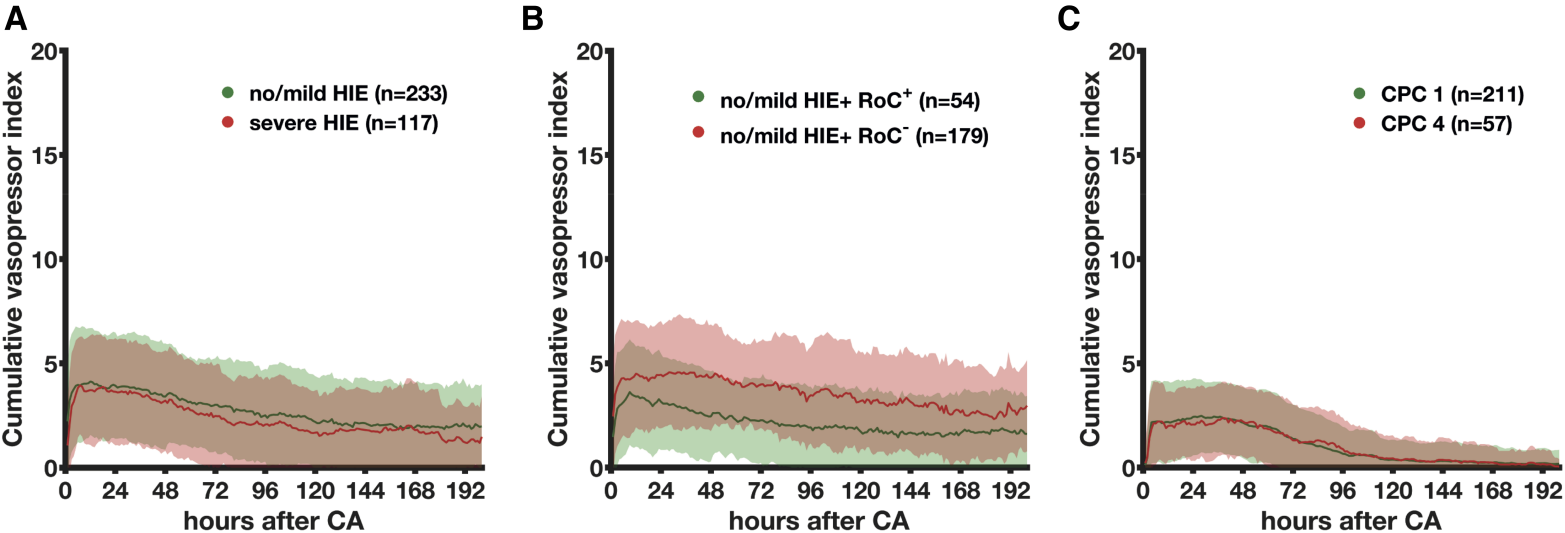
Association between the cumulative vasopressor index and the severity of hypoxic–ischemic encephalopathy evaluated by histopathology in non-survivors and neurological status in survivors. The association between the CVI as mean (bold line) with simple standard deviation (transparent area) and severity of HIE stratified by histopathology (**A**), regain of consciousness (**B**), and neurological status (**C**) is shown. MAP, mean arterial blood pressure; CA, cardiac arrest; HIE, hypoxic–ischemic encephalopathy; RoC^+^, regain of consciousness present; RoC^−^, never regained consciousness; CPC, cerebral performance category.

The vasopressor requirements differed between 233 no/mild HIE non-survivors and 117 severe HIE non-survivors (*p*_group _< 0.001) and time (*p* < 0.001), with a significant decrease (*p* = 0.01) observed in the severe HIE non-survivors between 72 and 96 h and a consequently higher CVI in no/mild HIE patients (Figure 3A). In the no/mild HIE non-survivors, regaining consciousness (*n* = 54, 23%) was associated with the lower (*p*_group _< 0.001) and faster (*p* < 0.001) decrease of vasopressors (Figure 3B). The CVI of the CPC 1 and CPC 4 patients both significantly decreased (*p* < 0.001) over time without a group difference (*p*_group _= 0.28) (Figure 3C). The group comparison showed that 211 CPC 1 patients and 57 CPC 4 patients had the lowest CVI (both mean, 1.0), followed by 54 no/mild HIE non-survivors with regain of consciousness (mean CVI, 2.1).

### Association between MAP and vasopressor therapy

Compared to the 233 no/mild HIE non-survivors, 117 severe HIE non-survivors required fewer vasopressors (CVI 2.3 vs. 2.7, *p* < 0.001) to achieve higher MAP values (mean, 73.1 vs. 72.0 mmHg, *p* = 0.001) (Supplementary Figure S1A). In these 233 no/mild HIE non-survivors, regain of consciousness (*n* = 54, 23%) was associated with higher MAP values (mean, 74.7 vs. 69.3 mmHg, *p* < 0.001) and fewer vasopressors (CVI 2.1 vs. 3.6, *p* < 0.001) (Supplementary Figure S1B). CPC 1 and CPC 4 patients needed the same vasopressor dosage (CVI 1.0) to achieve similar MAP values (mean, 81.2 vs. 82.3 mmHg, *p* = 0.10) (Supplementary Figure S1C). Supplementary Figure S2 and the results in the Supplementary Material show the influence of the 5-day survival. Notably, the no/mild HIE non-survivors with persistent coma had higher mean MAP values during the first 100 h in the case of the 5-day survival (71.1 vs. 67.1 mmHg).

### Association of death cause with vasopressor therapy and MAP

Of the 85 no/mild HIE non-survivors who suffered from cardiac death (Supplementary Figure S3), persistent coma (*n* = 50, 59%) was associated with a higher CVI (5.3 vs. 3.4, *p* < 0.001) and lower MAP values (mean, 64.8 vs. 69.8 mmHg, *p* < 0.001). Conversely, regain of consciousness (*n* = 12, 14%) was associated with a lower CVI (1.4 vs. 2.3, *p* < 0.001) and lower MAP (mean, 72.3 vs. 75.2 mmHg, *p* < 0.001). In all groups, MOF as a death cause (*n* = 158, 45%) was associated with higher vasopressor requirements (CVI ranging from 2.5 to 3.6), even in 30 (19%) no/mild HIE non-survivors who were regaining consciousness (CVI 2.5 vs. 1.6, *p* < 0.001). The MAP of 39 (11%) severe HIE non-survivors who suffered from MOF was lower compared to that for other death causes (mean, 68.6 vs. 75.1 mmHg, *p* < 0.001).

### Association of selected histopathological ND and clinically predicted brain injury on vasopressor therapy and MAP

**Figure 4:**
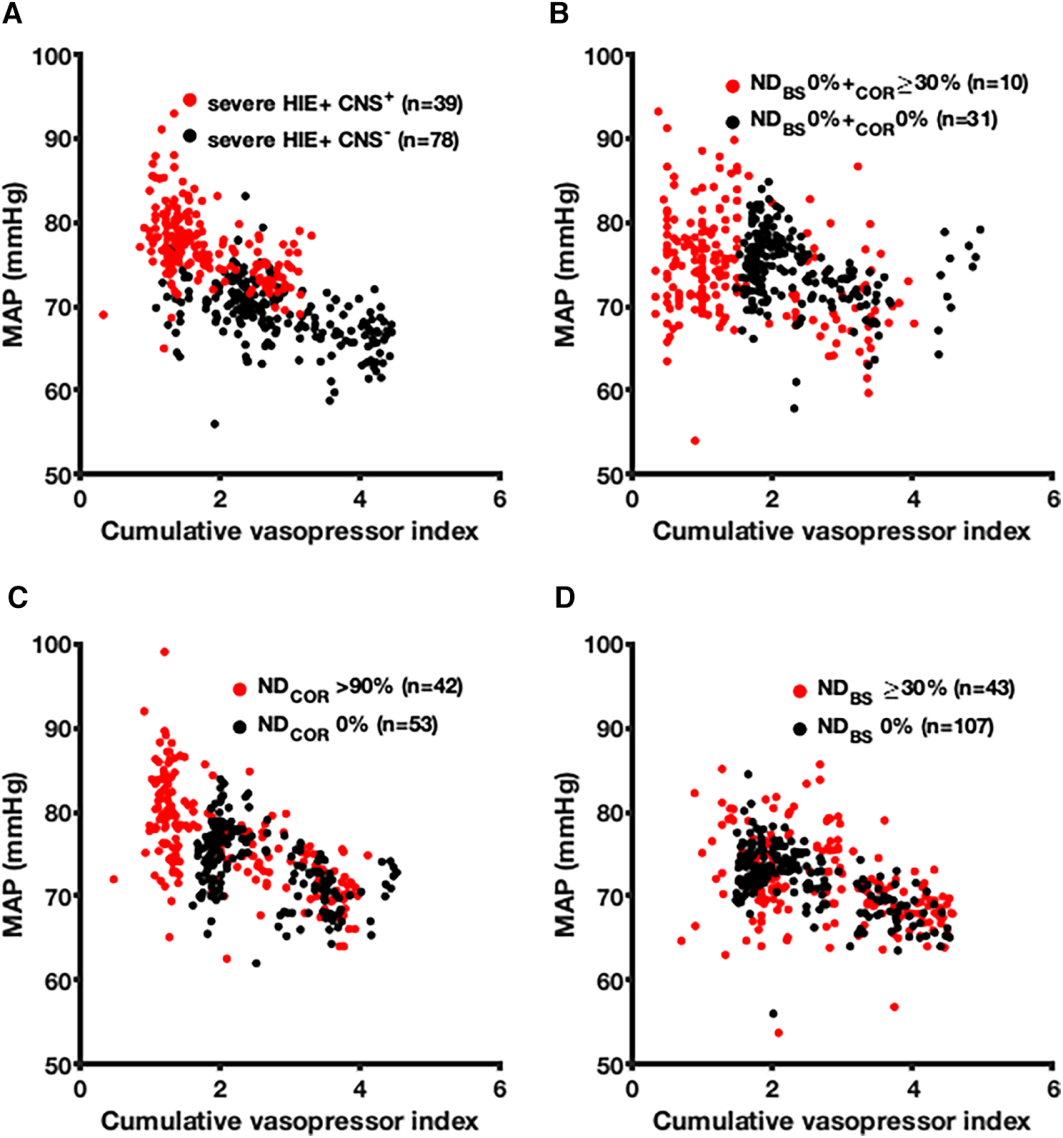
Association between the mean arterial blood pressure and the cumulative vasopressor index depending on the clinically predicted severe brain injury and neuronal death (ND) in the cerebral cortex and brainstem (BS) of non-survivors. The association between the MAP (hourly median value) and the CVI (hourly mean value) is illustrated depending on the clinically predicted severe brain injury and ND in the cerebral cortex and BS of non-survivors. (**A**) Clinically predicted presence (red) and absence (black) of severe brain injury studied in non-survivors. (**B**) Non-survivors with ND up from 30% in the cerebral cortex and without BS ND (red) compared to non-survivors without ND in both regions (black). (**C**) Cortical ND above 90% (red) compared to the absence of ND in the cerebral cortex (black). (**D**) ND up from 30% in the BS (red) compared to sparing of ND in the BS. MAP, mean arterial blood pressure; HIE, hypoxic–ischemic encephalopathy; CNS^+^, clinically predicted severe brain injury as death cause; CNS^−^, clinically predicted absence of severe brain injury; ND, neuronal death; BS, brainstem; COR, cerebral cortex.

In 39 (11%) severe HIE non-survivors with a clinically predicted brain injury as the death cause, the MAP was higher (mean, 69.2 vs. 77.2 mmHg, *p* < 0.001), and the CVI was lower (1.8 vs. 2.8, *p* < 0.001) compared to those with cardiac or MOF death causes (Figure 4A). To study selective cortical brain injury, we compared 41 non-survivors without brainstem (BS) ND showing at least 30% (*n* = 10, 24%) and no cortical ND (*n* = 31, 76%) (Figure 4B). The MAP values were indifferent (*p* = 0.57), but the CVI was higher in patients without cortical and BS ND (CVI 2.4 vs. 1.5, *p* < 0.001). In non-survivors with more than 90% cortical ND (*n* = 42, 44%) (Figure 4C), the CVI was lower (CVI 2.1 vs. 2.5, *p* < 0.001), and the MAP was higher (76.6 vs. 73.9 mmHg, *p* < 0.001) compared to those of 53 (56%) patients without cortical ND. Comparing the non-survivors without (*n* = 107, 71%) and more than 30% brainstem neuronal death (*n* = 43, 29%), we found no difference in the MAP (*p* = 0.12), but a lower CVI (2.5 vs. 2.7, *p* = 0.005) (Figure 4D). The group comparison showed that the non-survivors with more than 90% cortical ND had the highest MAP (mean, 76.6 mmHg), depicting a significant difference from all other five groups. The non-survivors with at least 30% cortical ND with BS sparing had the lowest CVI of 1.5.

## Discussion

Our study is hitherto the largest work performed to investigate the association between the MAP, vasopressors, and HIE evaluated through brain autopsy in non-survivors and two survivor cohorts unbiased by extracerebral death causes. Our main findings are as follows: (1) using histopathological and clinical outcomes, all cohorts with severe and no/mild HIE had mean MAP values above the guideline-recommended threshold of 65 mmHg; (2) non-survivors with a postmortem autopsy had lower mean MAP values (73.1 and 72.0 mmHg) than those of CPC 1 and CPC 4 survivors (81.2 and 82.3 mmHg, respectively); (3) evaluated by histopathology, there was no difference in the MAP values of severe and no/mild HIE non-survivors; (4) compared to persistent coma before death, regain of consciousness in no/mild HIE patients was associated with a higher MAP (74.7 vs. 69.3 mmHg); (5) coma awakening led to less and faster weaning of vasopressors, independent of the HIE severity; and (6) higher vasopressor requirements and lower MAP values were found in the absence of cortical ND, whereas BS ND was associated with higher vasopressor requirements.

In a systematic review (14), higher blood pressure was found to be associated with improved outcomes and lower mortality in patients with MAP values between 65 and 90 mmHg (13, 26, 27). Higher MAP values may counteract the post-ischemically impaired cerebral autoregulation, thereby avoiding secondary ischemia (9, 28, 29). However, interventional studies targeting higher MAP values of 72–100 mmHg compared to 65 mmHg failed to improve the outcome (15–18). Importantly, the clinical outcome was the primary endpoint in these studies, and death (i.e., CPC 5) mainly contributed to a poor outcome. In non-survivors, the absence of severe HIE and extracerebral death causes is frequent (1, 3). To bypass this limitation, we evaluated the HIE severity using histopathology and the clinical absence (CPC 1) and presence of HIE (CPC 4). Surprisingly, there was no MAP difference found between severe and no/mild HIE non-survivors. Both non-survivor groups showed a reversible MAP decrease during the first 48 h, similar to previous studies (30–32), where a poor outcome was correlated with early hypotension (31, 33–35).

Accordingly, compared to non-survivors, CPC 1 and CPC 4 survivors had higher MAP values without critical MAP drops. Hypotension during the dying process may explain the MAP differences between our non-survivor and survivor cohorts. CPC 4 survivors had significantly higher MAP values compared to CPC 1 survivors, but except for the first 24 h, the differences were not larger than 2 mmHg. Due to clinically proven severe HIE, the CPC 4 survivors enabled us to study—unbiased by death—the association between MAP and brain injury.

Considering death as a confounder of the HIE evaluation and based on the neurological status of the survivors, our results failed to show an association between MAP and brain injury; otherwise, we would have found higher MAP values in CPC 1 survivors compared to CPC 4 survivors and in no/mild HIE non-survivors. Importantly, the initial resuscitation time mainly affects the HIE severity, reflecting that CPC 4 and severe HIE non-survivors had the longest times compared to CPC 1 survivors and no/mild HIE non-survivors regaining consciousness. An animal study (36) corroborated that the resuscitation time determines the histopathological HIE severity in identical MAP regimes.

Extracerebral complications can cause prolonged coma and death despite the absence of HIE. To study this cohort, we dichotomized the no/mild HIE non-survivors into regain of consciousness and persistent coma before death. No/mild HIE non-survivors who regained consciousness showed stable blood pressure with a mean MAP of 74.7 mmHg. This result corroborates previous studies, in which even an hour of hypotension exposure is associated with higher mortality (30) and MAP stability during the first 48 h with higher survival, but without a better neurological outcome (37). Our mean MAP of 74.7 mmHg was higher compared to those in previous studies, in which 70 and 65 mmHg were the lower MAP thresholds for good neurological outcome (26) and survival (13), respectively. Unlike previous studies, we analyzed the MAP during the first 200 h and found a higher and stable MAP after 100 h, which may be explained by the increased sympathetic tone during awakening from coma (38, 39). Guided by near-infrared spectroscopy, intracranial pressure, and oxygen saturation, two small studies found MAP values of 89 (40) and 87–101 mmHg (29) to avoid secondary brain injury. In our study, persistently comatose non-survivors with histopathological no/mild HIE had a mean MAP of 69.3 mmHg, potentially supporting higher optimal MAP values. However, we cannot exclude regain of consciousness in the case of long-term survival.

Importantly, the association between the MAP and HIE cannot be studied without considering vasopressors. The CVI is an established tool for quantifying the vasopressor requirements (25) unaffected by TTM (41–43). Increased vasopressor requirement was associated with higher mortality (13, 32, 44). Accordingly, non-survivors had higher CVIs than CPC 1 and CPC 4 survivors, and their mean CVIs were similar to those in previous studies (41, 45). In no/mild HIE non-survivors, regain of consciousness was associated with a lower CVI without complete vasopressor weaning. A good outcome was previously more likely in patients with a MAP above 70 mmHg and vasopressor requirements similar to our CPC 1 and CPC 4 patients (26). However, excluding death as an HIE confounder, the vasopressor requirement of CPC 1 survivors was not different from that of CPC 4 survivors. Hence, our results contradict the statement that increased vasopressor requirements *per se* indicate a poor outcome in survivors with a MAP above 70 mmHg.

The incomplete vasopressor weaning in the no/mild HIE patients can be explained by extracerebral complications causing hemodynamic instability, prolonged vasopressor requirement, and death (1). Hence, our non-survivors who suffered from MOF death had a higher CVI, independent of the histopathological HIE severity (41, 46), and 5-day survival was associated with narrower CVI ranges indicating hemodynamic stability.

We found less vasopressor requirements in no/mild HIE non-survivors regaining consciousness, with higher MAP values reflecting the physiological response during coma awakening critically underlying BS control (38, 39). Accordingly, a selective brainstem neuronal death above 30% was associated with higher CVIs and unchanged MAP values compared to a histopathologically intact BS. The selective cortical ND was associated with less vasopressor requirements, independent of an additional BS injury. This corroborates the results of unresponsive wakefulness syndrome survivors showing less hemodynamic response and MAP stability to external stimuli (47, 48). Importantly, our results argue against the statement that selective cortical brain injury causes increased vasopressor requirements. By contrast, we found higher CVIs when the cortex showed no ND histopathologically.

This study had limitations. Due to the retrospective design, non-survivors with postmortem autopsies were limited and heterogeneous with a long inclusion period and inclusion from different ICUs. We cannot rule out selection biases for obtaining autopsies; hence, generalizability remains unknown. Importantly, the histopathological findings of our study are not easy to reproduce due to the limited number of postmortem autopsies in other centers. Furthermore, post-CA protocols have changed during the period including an increased accessibility to TTM. The SEND classification relies on a specific histopathological aspect of HIE using one staining. We recommend additional staining for synaptic damage and other cell types in future studies. Future histopathological studies should also analyze the interrater agreement and may consider using automated histopathological analyzing methods. Our negative result, i.e., no MAP difference between the severe and no/mild HIE groups, was strengthened by investigating two different cohorts. However, due to the non-interventional retrospective design of our study, we cannot exclude the possibility that a MAP effect on the outcome remains undetected due to confounding factors. We followed the international guideline of avoiding hypotension below 65 mmHg without doing a comparison to higher MAP targets. The histopathological validation of comparing MAP targets requires a prospective interventional trial and the standard operating procedure of vasopressor usage. Finally, our study focused on MAP, but blood gases like the mixed venous oxygen saturation, volume status, and microcirculation are other important hemodynamic parameters, as well as the usage of extracorporeal membrane oxygenation and other invasive hemodynamic devices.

## Conclusions

In a large retrospective study, we found lower MAP values in non-survivors with postmortem brain autopsies when compared to CPC 1 and CPC 4 survivors; however, all groups had MAP values above 65 mmHg. Based on a histopathological evaluation, there was no difference in the MAP of non-survivors with severe or no/mild HIE. Regain of consciousness was, however, associated with a higher MAP (74.7 vs. 69.3 mmHg) in no/mild HIE non-survivors compared to persistent coma before death. We found a lower requirement and a faster weaning of vasopressors in coma awakening, independent of the HIE severity. Our results provide no evidence related to elevating MAP targets above the current guideline recommendations to improve the neurological outcome after CA.

## Data Availability

The raw data supporting the conclusions of this article will be made available by the authors, without undue reservation.
