# Supplementary material for: Survival, but not the severity of hypoxic–ischemic encephalopathy, is associated with higher mean arterial blood pressure after cardiac arrest: a retrospective cohort study
Source: Front Cardiovasc Med. 2024 May 7;11:1337344. doi: 10.3389/fcvm.2024.1337344 (PMC11106407; doi:10.3389/fcvm.2024.1337344)
Supplement: Supplementary file 1 [file Datasheet1.pdf]

Supplementary Material

Survival, but not the severity of hypoxic–ischemic encephalopathy, is associated with higher mean arterial blood pressure after cardiac arrest: a retrospective cohort study

Sandra Preuß, MD[1,2], Jan Multmeier, MSc[1,3], Werner Stenzel, MD[4], Sebastian Major, MD[5], Christoph J. Ploner MD[1], Christian Storm, MD[6], Jens Nee, MD[6], Christoph Leithner, MD[1], Christian Endisch, MD[1]

[1]Department of Neurology, AG Emergency and Critical Care Neurology, Campus Virchow Klinikum, Charité Universitätsmedizin Berlin, Augustenburger Platz 1, 13353 Berlin, Germany

[2]Department of Cardiology and Angiology, Charité Campus Mitte, Charité Universitätsmedizin Berlin, Charitéplatz 1, 10117 Berlin, Germany

[3]Ada Health GmbH, Karl-Liebknecht-Str 1, 10178 Berlin, Germany

[4]Department of Neuropathology, Charité Campus Mitte, Charité Universitätsmedizin Berlin, Charitéplatz 1, 10117 Berlin, Germany

[5]Center for Stroke Research, Charité Universitätsmedizin Berlin, Charitéplatz 1, 10117 Berlin, Germany

[6]Department of Nephrology and Intensive Care Medicine, Cardiac Arrest Center of Excellence Berlin, Campus Virchow Klinikum, Charité Universitätsmedizin Berlin, Augustenburger Platz 1, 13353 Berlin, Germany

Corresponding author: Christian Endisch

Corresponding author's address: Department of Neurology, Campus Virchow Klinikum, Charité Universitätsmedizin Berlin, Augustenburger Platz 1, 13353 Berlin, Germany

Corresponding author's phone and fax: +49-30-450660384, fax +49-40-560902,

Corresponding author's e-mail address: christian.endisch@charite.de

Keywords: cardiac arrest; brain autopsy; hypoxic-ischemic encephalopathy, mean arterial pressure; cumulative vasopressor index; prognosis

Table of contents

Supplementary Table 1: Cumulative vasopressor index 3

Supplementary Figure 1: Association between mean arterial blood pressure, cumulative vasopressor index and severity of hypoxic-ischemic encephalopathy evaluated by histopathology in non-survivors and neurological status in survivors 4

Supplementary Figure 2: Association between mean arterial blood pressure, vasopressor usage and severity of hypoxic-ischemic encephalopathy evaluated by histopathology in non-survivors and neurological status in survivors with five-day survival 5

Supplementary Figure 3: Association between mean arterial blood pressure and cumulative vasopressor index stratified by histopathological severity of hypoxic-ischemic encephalopathy, regain of consciousness and death causes in non-survivors 6

Supplementary Results: Cumulative vasopressor index and mean arterial pressure in patients with five-day survival 7

Supplementary Table 2: Preexisting diseases of patients 8

Supplementary Table 3: Non-survivor characteristics stratified by death causes and regain of consciousness 9

Supplementary Table 4: Preexisting diseases of non-survivors stratified by death causes and regain of consciousness 10

	**Cumulative vasopressor index 1 point**	**Cumulative vasopressor index 2 points**	**Cumulative vasopressor index 3 points**	**Cumulative vasopressor index 4 points**
**Vasopressor**	**dosage**	**dosage**	**dosage**	**dosage**
Vasopressin (units/min)	-	-	-	any
Norepinephrine (µg/kg/min)	-	0 < dose ≤ 0.05	0.05 < dose ≤ 0.1	> 0.1
Epinephrine (µg/kg/min)	-	0 < dose ≤ 0.05	0.05 < dose ≤ 0.1	> 0.1
Phenylephrine (µg/kg/min)	-	0 < dose ≤ 0.4	0.4 < dose ≤ 0.8	> 0.8
Dopamine (µg/kg/min)	0 < dose ≤ 5	5 < dose ≤ 10	10 < dose ≤ 15	> 15

Supplementary Table 1. Cumulative vasopressor index.

To quantify the vasopressor requirements, we used the cumulative vasopressor index (CVI). Depending on the current dosages of the used vasoactive drugs, the CVI allocates points and thus quantifies the current vasopressor need at any time point.

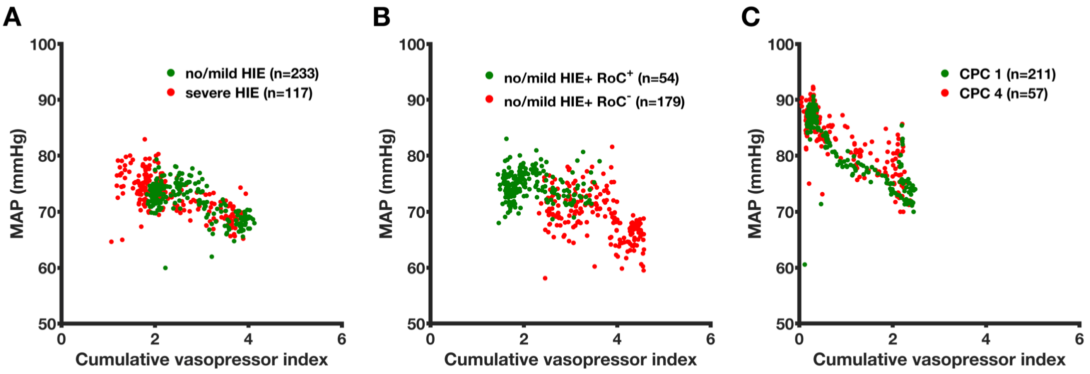

Supplementary Figure 1: Association between mean arterial blood pressure, cumulative vasopressor index and severity of hypoxic-ischemic encephalopathy evaluated by histopathology in non-survivors and neurological status in survivors.

This figure shows the association between hourly median MAP and hourly mean CVI depending on histopathological severity of HIE (A), regain of consciousness (B) and clinical absence of HIE (C). MAP – mean arterial blood pressure, HIE – hypoxic-ischemic encephalopathy, RoC^+ – regain of consciousness present, RoC^- – never regained consciousness, CPC – cerebral performance category

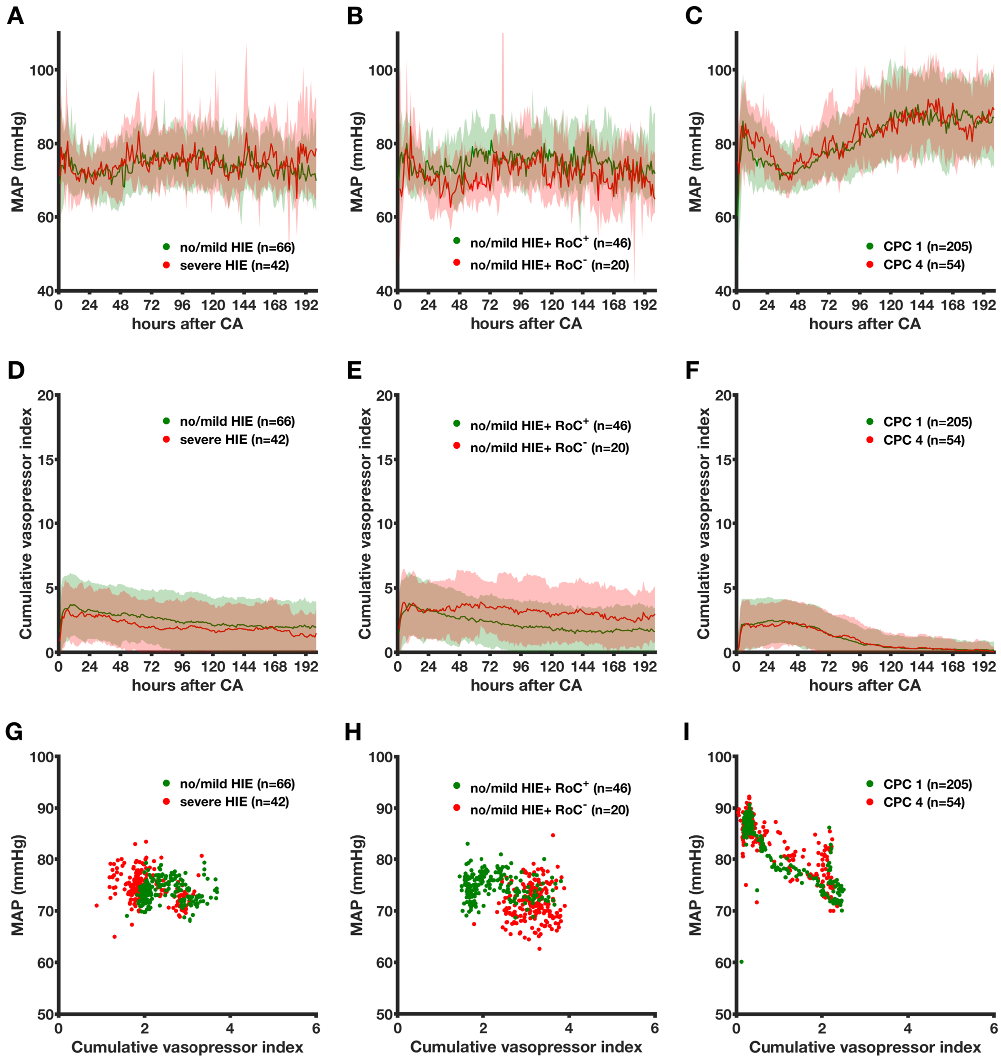

Supplementary Figure 2: Association between mean arterial blood pressure, vasopressor usage and severity of hypoxic-ischemic encephalopathy evaluated by histopathology in non-survivors and neurological status in survivors with five-day survival.

To exclude prolonged dying processes as a confounder, we separately studied non-survivors and survivors with five-day survival. In the first row, MAP is illustrated as median (bold line) and interquartile range (transparent area) depending on histopathological HIE severity (A), regain of consciousness (B) and clinically absent HIE (C). The second row shows the association between CVI as mean (bold line) with simple standard deviation (transparent area) and HIE severity stratified by histopathology (D), regain of consciousness (E) and clinical outcome (F). The association between MAP (hourly median value) and CVI (hourly mean value) is illustrated in the third row (G – I). MAP – mean arterial blood pressure, CA – cardiac arrest, HIE – hypoxic-ischemic encephalopathy, RoC^{+} – regain of consciousness present, RoC^{-} – never regained consciousness, CPC – cerebral performance category

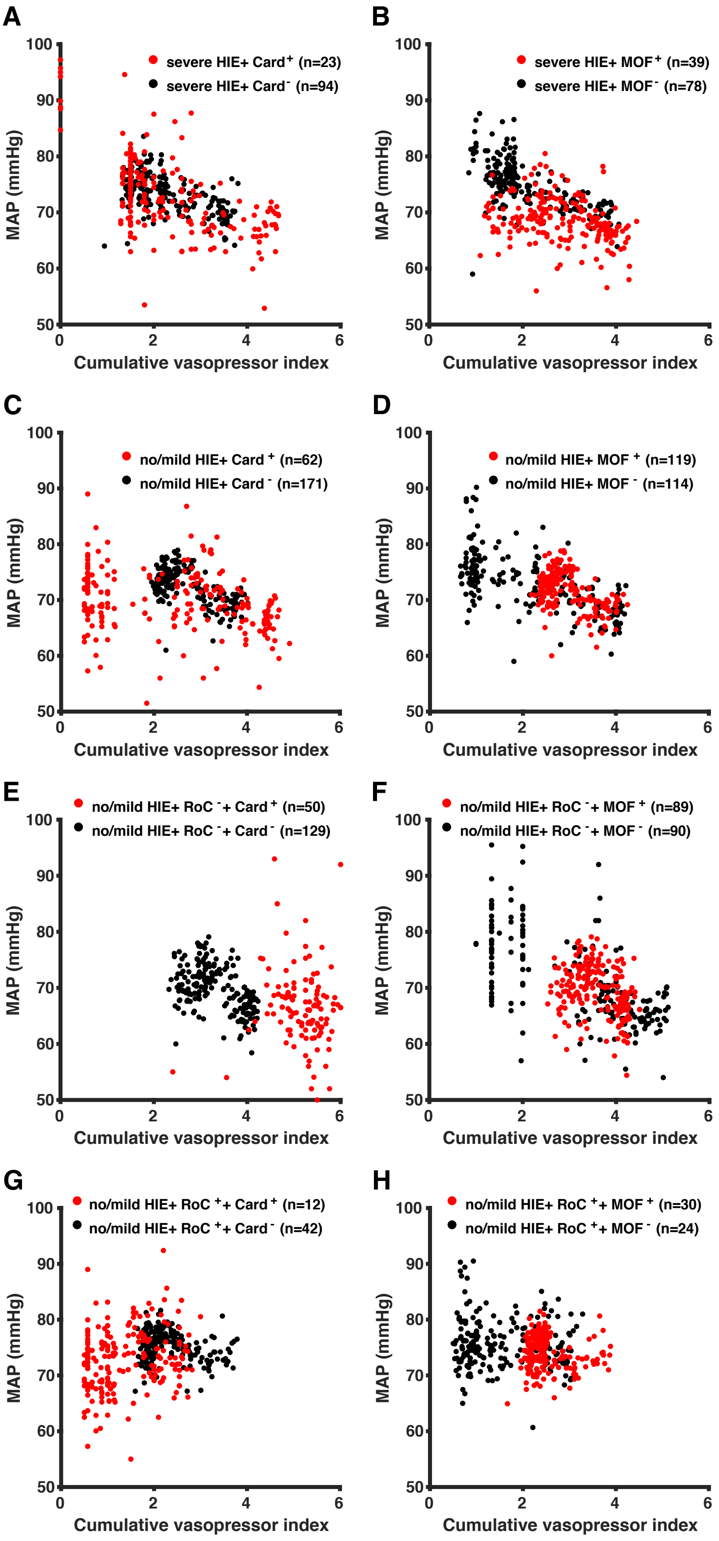

Supplementary Figure 3: Association between mean arterial blood pressure and cumulative vasopressor index stratified by histopathological severity of hypoxic-ischemic encephalopathy, regain of consciousness and death causes in non-survivors.

In each figure, the association between MAP (hourly median value) and CVI (hourly mean value) is illustrated depending on histopathological HIE severity, regain of consciousness and death cause. Death causes include cardiac (A, C, E, G), and sepsis/multiorgan failure (B, D, F, H) and were stratified by severe HIE (A, B), no/mild HIE (C, D), no/mild HIE without regain of consciousness (E, F) and no/mild HIE with temporary regain of consciousness (G, H). MAP – mean arterial blood pressure, HIE – hypoxic-ischemic encephalopathy, Card$^+$ – cardiac death cause present, Card$^-$ – cardiac death cause not present, MOF$^+$ – sepsis/multiorgan failure present, MOF$^-$ – sepsis/multiorgan failure not present, CA – cardiac arrest

Supplementary Results: Cumulative vasopressor index and mean arterial pressure in patients with five-day survival.

To investigate the confounder of death-related hypotension and hemodynamic instability during the early post-CA period, we separately studied the association between MAP, vasopressor usage and severity of HIE in patients with five-days survival (Supplemental Figure 1). Results of MAPs remained unchanged comparing no/mild (n=66) and severe HIE non-survivors (n=42), respectively, CPC 1 (n=205) and CPC 4 survivors (n=54). The MAP of no/mild HIE non-survivors with (n=46) and without (n=20) regain of consciousness was different (p_{group}<0.001) but showed no differences over time (p=0.51). No/mild HIE non-survivors with five-day survival had a mean MAP of 73.8 mmHg compared to 72.0 mmHg in all no/mild HIE non-survivors. Notably, mean MAP of no/mild HIE non-survivors without regain of consciousness during the first 100 hours was higher in the cohort surviving at least five days (71.1 mmHg vs 67.1 mmHg) and the reversible early MAP drop remained significant (p=0.006 during 48 – 72, p=0.002 during 72 – 96, and p=0.002 during 96 – 120 hours). During the 200-hour period, MAP of no/mild HIE non-survivors without regain of consciousness was higher and within a narrower range (mean 71.5 mmHg, IQR 69.0 – 74.0, SD 3.5) compared to patients surviving less than five days (mean 69.3 mmHg, IQR 66.3 – 72.0, SD 4.2). Vasopressor requirements decreased significantly faster during a longer period in severe HIE non-survivors compared to no/mild HIE non-survivors between 48 – 72 (p=0.003) and 72 – 96 hours (p=0.012). In 46 no/mild HIE non-survivors with regain of consciousness, vasopressor requirements were lower (p<0.001) and decreased faster (p<0.001) apart from the first 24 hours. Severe HIE non-survivors surviving five days had a lower mean CVI during the first 100 hours (CVI 3.4 vs 4.1). Results of vasopressor requirements for CPC 1 and 4 survivors remained unchanged. Patients with five-day survival had narrower range of vasopressor requirements with a mean CVI of 2.5 (IQR 2.1 – 2.9, SD 0.5 vs mean CVI 2.7, IQR 2.1 – 3.4, SD 0.7) in no/mild HIE non-survivors and a mean CVI of 2.0 (IQR 1.7 – 2.4, SD 0.5 vs mean CVI 2.3, IQR 1.7 – 3.0, SD 0.8) in severe HIE non-survivors. No/mild HIE non-survivors without regain of consciousness had also narrower ranges and lower requirements of vasopressor in case of five-day survival (mean CVI of 3.2, IQR 2.9 – 3.4, SD 0.4 vs mean CVI 3.6, IQR 3.0 – 4.2, SD 0.7). In no/mild HIE non-survivors with regain of consciousness, the range of vasopressor requirements was unchanged by survival days (mean CVI 2.2, IQR 1.7 – 2.6, SD 0.6 vs mean CVI 2.1, IQR 1.7 – 2.5, SD 0.6).

Supplementary Table 2: Preexisting diseases of patients.

Demographic characteristics	severe HIE non-survivors (n=117)	no/mild HIE non-survivors (n= 233)	Statistical comparison severe HIE vs. no/mild HIE non-survivors, test statistic (p)[1]	CPC 4 survivors (n=57)	CPC 1 survivors (n=211)	Statistical comparison CPC 4 vs. CPC 1 survivors, test statistic (p)[1]
**Acute myocardial infarction, (n, %)**	28 (24)	48 (21)	$\chi^2 = 0.33$ ($p = 0.565$)	18 (32)	96 (46)	$\chi^2 = 3.010$ ($p = 0.083$)
**Chronic heart failure, (n, %)**	65 (56)	133 (57)	$\chi^2 = 0.025$ ($p = 0.875$)	32 (56)	166 (79)	$\chi^2 = 10.669$ ($p = .001$)
**Cardiomyopathy, (n, %)**	4 (3)	19 (8)	$\chi^2 = 2.126$ ($p = 0.145$)	5 (9)	26 (12)	$\chi^2 = 0.260$ ($p = 0.610$)
**Chronic ischemic cardiomyopathy, (n, %)**	48 (41)	84 (36)	$\chi^2 = 0.622$ ($p = 0.430$)	23 (40)	144 (68)	$\chi^2 = 13.706$ ($p < 0.001$)
**Ischemic cardiomyopathy, (n, %)**	9 (8)	19 (8)	$\chi^2 < 0.001$ ($p > 0.999$)	1 (2)	19 (9)	$\chi^2 = 2.447$ ($p = 0.118$)
**Chronic ischemic cardiomyopathy, Atheromatosis without significant stenosis, (n, %)**	0 (0)	4 (2)	$\chi^2 = 0.796$ ($p = 0.372$)	0 (0)	5 (2)	$\chi^2 = 0.386$ ($p = 0.534$)
**Chronic ischemic cardiomyopathy, 1-vessel disease, (n, %)**	12 (10)	12 (5)	$\chi^2 = 2.430$ ($p = 0.119$)	4 (7)	39 (19)	$\chi^2 = 3.57$ ($p = 0.059$)
**Chronic ischemic cardiomyopathy, 2-vessel disease, (n, %)**	10 (9)	11 (5)	$\chi^2 = 1.401$ ($p = 0.237$)	6 (11)	41 (19)	$\chi^2 = 15.98$ ($p < 0.001$)
**Chronic ischemic cardiomyopathy, 3-vessel disease, (n, %)**	21 (18)	48 (21)	$\chi^2 = 0.199$ ($p = 0.656$)	11 (19)	66 (31)	$\chi^2 = 1.884$ ($p = 0.170$)
**Other cardiac arrhythmias, (n, %)**	34 (29)	51 (22)	$\chi^2 = 1.806$ ($p = 0.179$)	21 (37)	137 (65)	$\chi^2 = 13.492$ ($p < 0.001$)
**Atrial fibrillation and flutter, (n, %)**	47 (40)	94 (40)	$\chi^2 < 0.001$ ($p > 0.999$)	18 (32)	53 (25)	$\chi^2 = 0.659$ ($p = 0.417$)
**Chronic renal failure, (n, %)**	31 (27)	55 (24)	$\chi^2 = 0.212$ ($p = 0.645$)	1 (2)	38 (18)	$\chi^2 = 8.274$ ($p = 0.004$)
**Chronic obstructive pulmonary disease, (n, %)**	24 (21)	51 (22)	$\chi^2 = 0.025$ ($p = 0.875$)	12 (21)	28 (13)	$\chi^2 = 1.572$ ($p = 0.21$)
**Diabetes mellitus Type 2 (n, %)**	33 (28)	61 (26)	$\chi^2 = 0.076$ ($p = 0.783$)	17 (30)	35 (17)	$\chi^2 = 4.217$ ($p = 0.040$)
**Alcoholic liver disease, (n, %)**	1 (1)	15 (6)	$\chi^2 = 4.359$ ($p = 0.037$)	0 (0)	3 (1)	$\chi^2 = 0.038$ ($p = 0.845$)
**Morbid obesity, (n, %)**	7 (6)	15 (6)	$\chi^2 < 0.001$ ($p > 0.999$)	0 (0)	8 (4)	$\chi^2 = 1.111$ ($p = 0.292$)
**Arterial hypotension, (n, %)**	6 (5)	11 (5)	$\chi^2 = 0.106$ ($p = 0.745$)	1 (2)	3 (1)	$\chi^2 < 0.001$ ($p = 0.979$)
**Nonrheumatic aortic valve stenosis, (n, %)**	9 (8)	15 (6)	$\chi^2 = 0.045$ ($p = 0.831$)	1 (2)	1 (1)	$\chi^2 = 0.017$ ($p = 0.897$)
**Nonrheumatic aortic valve insufficiency, (n, %)**	3 (3)	10 (4)	$\chi^2 = 0.257$ ($p = 0.612$)	1 (2)	4 (2)	$\chi^2 < 0.001$ ($p > 0.999$)

[1]Categorical variables were compared using Chi-square tests (χ^2). HIE – hypoxic-ischemic encephalopathy, CPC – cerebral performance category

Supplementary Table 3: Non-survivor characteristics stratified by death causes and regain of consciousness.

Demogaphic characteristics	Cardiac death cause (n=85)	Statistical comparison Cardiac death cause vs. non-cardiac death cause, test statistic (*p*)[1]	MOF death cause (n=158)	Statistical comparison MOF death cause vs. non-MOF death cause, test statistic (*p*)[1]	CNS death cause (n=41)	Statistical comparison CNS death cause vs. non-CNS death cause, test statistic (*p*)[1]	no/mild HIE non-survivors + conscious (n= 54)	no/mild HIE non-survivors + never conscious (n= 179)	Statistical comparison no/mild HIE non-survivors + conscious vs. no/mild HIE non-survivors + never conscious, test statistic (*p*)[1]
**Gender, male (n, %)**	50 (59)	$\chi^2 = 0.1416$ ($p = 0.707$)	97 (61)	$\chi^2 < 0.001$ ($p > 0.999$)	26 (63)	$\chi^2 = 0.0216$ ($p = 0.883$)	34 (63)	111 (62)	$\chi^2 < 0.001$ ($p > 0.999$)
**Age, year (median, IQR)**	73 (64 – 79)	W = 14084 ($p < 0.001$)	68 (58 – 75)	W = 14782 ($p = 0.682$)	63 (59 – 73)	W = 5680 ($p = 0.283$)	68 (58 – 77)	69 (58 – 76)	W = 4884.5 ($p = 0.906$)
**OHCA, (n, %)**	68 (80)	$\chi^2 = 0.4059$ ($p = 0.524$)	145 (92)	$\chi^2 = 14.992$ ($p < 0.001$)	20 (49)	$\chi^2 = 35.297$ ($p < 0.001$)	51 (94)	164 (92)	$\chi^2 = 0.153$ ($p = 0.696$)
**Cardiac cause of CA, (%)**	78	$\chi^2 = 75.569$ ($p < 0.001$)	17	$\chi^2 = 24.336$ ($p < 0.001$)	39	$\chi^2 = 0.319$ ($p = 0.573$)	34	30	χ^2 =0.105 (p=0.745)
**Shockable initial rhythm, (%)**	39	$\chi^2 = 7.857$ ($p = 0.005$)	20	$\chi^2 = 4.021$ ($p = 0.044$)	35	$\chi^2 = 1.455$ ($p = 0.228$)	22	24	$\chi^2 < 0.001$ ($p > 0.999$)
**tROSC, min, (median, IQR)**	10 (8 – 25)	W = 6035 ($p = 0.365$)	10 (4 – 20)	W = 5679 ($p < 0.001$)	20 (15 – 25)	W = 3816.5 ($p = 0.365$)	5 (1 – 10)	11 (5 – 27)	W = 1713.5 ($p < 0.001$)
**Targeted temperature management, (n, %)**	16 (19)	$\chi^2 = 2.950$ ($p = 0.086$)	34 (22)	$\chi^2 = 3.311$($p = 0.069$)	29 (71)	$\chi^2 = 43.889$ ($p < 0.001$)	9 (17)	31 (17)	$\chi^2 < 0.001$ ($p > 0.999$)
Second CA with resuscitation during ICU stay, (n, %)	56 (66)	$\chi^2 = 18.956$ ($p < 0.001$)	60 (38)	$\chi^2 = 5.02$ ($p = 0.025$)	8 (20)	$\chi^2 = 10.928$ ($p < 0.001$)	24 (44)	84 (47)	$\chi^2 = 0.027$ ($p = 0.869$)
Temporarily conscious during ICU stay, (n, %)	15 (18)	$\chi^2 = 0.155$ ($p = 0.694$)	37 (23)	$\chi^2 = 2.088$ ($p = 0.149$)	6 (15)	$\chi^2 = 0.437$ ($p = 0.508$)	54 (100)	0 (0)	$\chi^2 = 227.42$ ($p < 0.001$)
WLST, (n, %)	19 (22)	$\chi^2 = 19.689$ ($p < 0.001$)	69 (44)	$\chi^2 < 0.001$ ($p > 0.999$)	40 (98)	$\chi^2 = 52.275$ ($p < 0.001$)	32 (59)	59 (33)	$\chi^2 = 10.974$ ($p < 0.001$)
**Length of ICU stay, day (median, IQR)**	1 (0 – 3)	W = 8552.5 ($p < 0.001$)	3 (1 – 18)	W = 17848.5 ($p =$ 0.004)	7 (3 - 11)	W = 8550.5 ($p < 0.001$)	25 (9 – 53)	1 (0 – 3)	W = 8841.5 ($p < 0.001$)
Death cause, cardiac, (n, %)	85 (100)	-	0 (0)	-	0 (0)	-	*12 (22)*	*50 (28)*	$\chi^2 = 0.431$ ($p = 0.511$)
Death cause, MOF, (n, %)	0 (0)	-	158 (100)	-	0 (0)	-	*30 (56)*	*89 (50)*	$\chi^2 = 0.356$ ($p = 0.551$)
Death cause, CNS, (n, %)	0 (0)	-	0 (0)	-	41 (100)	-	*1 (2)*	*1 (1)*	$\chi^2 = 0.004$ ($p = 0.951$)
Death cause, others, (n, %)	0 (0)	-	0 (0)	-	0 (0)	-	*11 (20)*	*39 (22)*	$\chi^2 = 0.001$ ($p = 0.974$)
no/mild HIE, yes=1 (n, %)	62 (73)	$\chi^2 = 1.686$ ($p = 0.194$)	119 (75)	$\chi^2 = 9.195$ ($p = 0.002$)	2 (5)	$\chi^2 = 76.317$ ($p < .001$)	54 (100)	179 (100)	-
Maximal SEND score (range 0 – 4), cortex, (median, IQR)	1 (1 – 1)	W = 10348 ($p = 0.211$)	1 (1 – 1)	W = 12306 ($p < 0.001$)	4 (2 – 4)	W = 10952 ($p < .001$)	1 (0 – 1)	1 (1 – 1)	W = 4167 ($p = 0.035$)
Maximal SEND score (range 0 – 4), hippocampus, (median, IQR)	1 (1 – 2)	W = 9445.5 ($p = 0.063$)	1 (1 – 2)	W = 11880 ($p = 0.003$)	4 (3 – 4)	W = 10952 ($p < .001$)	1 (1 – 1)	1 (1 – 1)	W = 4546 ($p = 0.777$)
Maximal SEND score (range 0 – 4), cerebellum, (median, IQR)	1 (0 – 1)	W = 8572 ($p = 0.033$)	1 (0 – 1)	W = 12386 ($p = 0.119$)	4 (2 – 4)	W = 9160 ($p < .001$)	1 (0 – 1)	1 (1 – 1)	W = 4850 ($p = 0.433$)
Maximal SEND score (range 0 – 4), brainstem, (median, IQR)	1 (0 – 1)	W = 9535 ($p = 0.043$)	1 (0 – 1)	W = 14495 ($p = 0.749$)	1 (1 – 4)	W = 8971 ($p < .001$)	1 (0 – 1)	1 (0 – 1)	W = 4908 ($p = 0.782$)

[1]Categorical variables were compared using Chi-square tests (χ^2), non-normally distributed continuous variables were compared using Wilcoxon rank sum tests (W). MOF – sepsis/multiorgan failure, CNS – clinically predicted severe brain injury, HIE – hypoxic-ischemic encephalopathy, IQR – inter-quartile range, OHCA – out-of-hospital cardiac arrest, CA – cardiac arrest, tROSC – time from cardiac arrest to spontaneous circulation, ICU – intensive care unit, WLST – withdrawal of life-sustaining treatment, SEND – selective eosinophilic neuronal death

Supplementary Table 4: Preexisting diseases of non-survivors stratified by death causes and regain of consciousness.

Demographic characteristics	Cardiac death cause (n=85)	Statistical comparison Cardiac death cause vs. non-cardiac death cause, test statistic (p)[1]	MOF death cause (n=158)	Statistical comparison MOF death cause vs. non-MOF death cause, test statistic (p)[1]	CNS death cause (n=41)	Statistical comparison CNS death cause vs. non-CNS death cause, test statistic (p)[1]	no/mild HIE non-survivors + conscious (n= 54)	no/mild HIE non-survivors + never conscious (n= 179)	Statistical comparison no/mild HIE non-survivors + conscious vs. no/mild HIE non-survivors + never conscious, test statistic (p)[1]
**Acute myocardial infarction, (n, %)**	30 (35)	$\chi^2 < 0.001$ ($p = 0.979$)	59 (37)	$\chi^2 = 0.131$ ($p = 0.717$)	20 (49)	$\chi^2 = 2.694$ ($p = 0.101$)	20 (37)	62 (35)	$\chi^2 = 0.026$ ($p = 0.872$)
**Chronic heart failure, (n, %)**	35 (41)	$\chi^2 = 23.525$ ($p < 0.001$)	23 (15)	$\chi^2 = 7.929$ ($p = 0.717$)	8 (20)	$\chi^2 = 0.026$ ($p = 0.871$)	10 (19)	38 (21)	$\chi^2 = 0.06$ ($p = 0.811$)
**Cardiomyopathy, (n, %)**	66 (78)	$\chi^2 = 19.18$ ($p < 0.001$)	83 (53)	$\chi^2 = 1.625$ ($p = 0.202$)	20 (49)	$\chi^2 = 0.816$ ($p = 0.366$)	35 (65)	98 (55)	$\chi^2 = 1.330$ ($p = 0.249$)
**Chronic ischemic cardiomyopathy, (n, %)**	7 (8)	$\chi^2 = 0.212$ ($p = 0.646$)	12 (8)	$\chi^2 = 0.235$ ($p = 0.628$)	1 (2)	$\chi^2 = 0.642$ ($p = 0.423$)	4 (7)	15 (8)	$\chi^2 < 0.001$ ($p > 0.999$)
**Ischemic cardiomyopathy, (n, %)**	53 (62)	$\chi^2 = 27.643$ ($p < 0.001$)	45 (29)	$\chi^2 = 9.749$ ($p = 0.002$)	19 (46)	$\chi^2 = 1.085$ ($p = 0.298$)	17 (32)	67 (37)	$\chi^2 = 0.405$ ($p = 0.525$)
**Chronic ischemic cardiomyopathy, Atheromatosis without significant stenosis, (n, %)**	16 (19)	$\chi^2 = 15.98$ ($p < 0.001$)	6 (4)	$\chi^2 = 5.98$ ($p < 0.001$)	5 (12)	$\chi^2 = 0.559$ ($p = 0.455$)	4 (7)	15 (8)	$\chi^2 < 0.001$ ($p > 0.999$)
**Chronic ischemic cardiomyopathy, 1-vessel disease, (n, %)**	1 (1)	$\chi^2 < 0.001$ ($p > 0.999$)	2 (1)	$\chi^2 < 0.001$ ($p > 0.999$)	0 (0)	$\chi^2 < 0.001$ ($p > 0.999$)	1 (2)	3 (2)	$\chi^2 < 0.001$ ($p > 0.999$)
**Chronic ischemic cardiomyopathy, 2-vessel disease, (n, %)**	8 (9)	$\chi^2 = 0.680$ ($p = 0.410$)	6 (4)	$\chi^2 = 3.396$ ($p = 0.065$)	5 (12)	$\chi^2 = 1.233$ ($p = 0.267$)	3 (6)	9 (5)	$\chi^2 < 0.001$ ($p > 0.999$)
**Chronic ischemic cardiomyopathy, 3-vessel disease, (n, %)**	6 (7)	$\chi^2 = 15.98$ ($p < 0.001$)	7 (4)	$\chi^2 = 0.802$ ($p < 0.371$)	7 (17)	$\chi^2 = 7.995$ ($p = 0.005$)	3 (6)	8 (5)	$\chi^2 < 0.001$ ($p > 0.999$)
**Other cardiac arrhythmias, (n, %)**	0 (0)	$\chi^2 = 0.100$ ($p = 0.757$)	2 (1)	$\chi^2 = 0.029$ ($p = 0.865$)	0 (0)	$\chi^2 < 0.001$ ($p > 0.999$)	0 (0)	3 (2)	$\chi^2 = 0.072$ ($p = 0.788$)
**Atrial fibrillation and flutter, (n, %)**	29 (34)	$\chi^2 = 5.217$ ($p = 0.022$)	30 (19)	$\chi^2 = 3.888$ ($p = 0.049$)	14 (34)	$\chi^2 = 1.886$ ($p = 0.170$)	14 (26)	37 (21)	$\chi^2 = 0.398$ ($p = 0.528$)
**Chronic renal failure, (n, %)**	4 (5)	$\chi^2 = 0.004$ ($p = 0.949$)	4 (3)	$\chi^2 = 0.995$ ($p = 0.319$)	5 (12)	$\chi^2 = 5.885$ ($p = 0.015$)	3 (6)	5 (3)	$\chi^2 = 0.303$ ($p = 0.582$)
**Chronic obstructive pulmonary disease, (n, %)**	32 (38)	$\chi^2 = 9.445$ ($p = 0.002$)	34 (22)	$\chi^2 = 1.163$ ($p = 0.281$)	10 (24)	$\chi^2 < 0.001$ ($p > 0.999$)	14 (26)	41 (23)	$\chi^2 = 0.076$ ($p = 0.783$)
**Diabetes mellitus Typ 2 (n, %)**	12 (14)	$\chi^2 = 3.014$ ($p = 0.083$)	41 (26)	$\chi^2 = 3.024$ ($p = 0.082$)	12 (29)	$\chi^2 = 1.209$ ($p = 0.272$)	11 (20)	40 (22)	$\chi^2 = 0.014$ ($p = 0.904$)
**Alcoholic liver disease, (n, %)**	12 (14)	$\chi^2 = 7.122$ ($p = 0.008$)	57 (36)	$\chi^2 = 15.215$ ($p < 0.001$)	7 (17)	$\chi^2 = 1.139$ ($p = 0.247$)	13 (24)	51 (29)	$\chi^2 = 0.215$ ($p = 0.643$)
**Morbid obesity, (n, %)**	1 (1)	$\chi^2 = 2.027$ ($p = 0.155$)	11 (7)	$\chi^2 = 2.840$ ($p = 0.091$)	0 (0)	$\chi^2 = 1.296$ ($p = 0.274$)	3 (6)	12 (7)	$\chi^2 < 0.001$ ($p > 0.999$)
**Arterial hypotension, (n, %)**	7 (8)	$\chi^2 = 0.353$ ($p = 0.552$)	9 (6)	$\chi^2 = 0.036$ ($p = 0.849$)	5 (12)	$\chi^2 = 1.734$ ($p = 0.188$)	4 (7)	11 (6)	$\chi^2 < 0.001$ ($p = 0.988$)
**Nonrheumatic aortic valve stenosis, (n, %)**	5 (6)	$\chi^2 = 0.046$ ($p = 0.830$)	10 (6)	$\chi^2 = 0.832$ ($p = 0.361$)	1 (2)	$\chi^2 = 0.144$ ($p = 0.704$)	0 (0)	11 (6)	$\chi^2 = 2.251$ ($p = 0.134$)
**Nonrheumatic aortic valve insufficiency, (n, %)**	12 (14)	$\chi^2 = 7.825$ ($p = 0.005$)	10 (6)	$\chi^2 = 0.020$ ($p = 0.887$)	0 (0)	$\chi^2 = 2.311$ ($p = 0.129$)	5 (9)	10 (6)	$\chi^2 = 0.419$ ($p = 0.517$)

[1]Categorical variables were compared using Chi-square tests (χ^2), non-normally distributed continuous variables were compared using Wilcoxon rank sum tests (W). MOF – sepsis/multiorgan failure, CNS – clinically predicted severe brain injury, HIE – hypoxic-ischemic encephalopathy, IQR – inter-quartile range, OHCA – out-of-hospital cardiac arrest, CA – cardiac arrest, tROSC – time from cardiac arrest to spontaneous circulation, ICU – intensive care unit, WLST – withdrawal of life-sustaining treatment, SEND – selective eosinophilic neuronal death
